# Nuclear Receptors in Asthma: Empowering Classical Molecules Against a Contemporary Ailment

**DOI:** 10.3389/fimmu.2020.594433

**Published:** 2021-01-26

**Authors:** Drishti Tiwari, Pawan Gupta

**Affiliations:** Department of Molecular Biology, Council of Scientific and Industrial Research, Institute of Microbial Technology, Chandigarh, India

**Keywords:** asthma, comorbidities, glucocorticoids, diet, nuclear receptors

## Abstract

The escalation in living standards and adoption of ‘Western lifestyle’ has an allied effect on the increased allergy and asthma burden in both developed and developing countries. Current scientific reports bespeak an association between allergic diseases and metabolic dysfunction; hinting toward the critical requirement of organized lifestyle and dietary habits. The ubiquitous nuclear receptors (NRs) translate metabolic stimuli into gene regulatory signals, integrating diet inflences to overall developmental and physiological processes. As a consequence of such promising attributes, nuclear receptors have historically been at the cutting edge of pharmacy world. This review discusses the recent findings that feature the cardinal importance of nuclear receptors and how they can be instrumental in modulating current asthma pharmacology. Further, it highlights a possible future employment of therapy involving dietary supplements and synthetic ligands that would engage NRs and aid in eliminating both asthma and linked comorbidities. Therefore, uncovering new and evolving roles through analysis of genomic changes would represent a feasible approach in both prevention and alleviation of asthma.

## Introduction

The advancement of science has caused a reduction in both mortality and morbidity associated with infectious diseases. However, the modern lifestyle has constituted a world where cases of metabolic and allergic diseases are prolific. The incidences of allergic diseases constitute the most chronic maladies globally, with asthma leading the class. Asthma equally affects people from different socioeconomic status or age groups and accounts for 339 million affected people worldwide (1). An increase in allergic disorders has also been observed with the adoption of sedentary lifestyle and higher living standards. The condition further deteriorates due to the prevalence of comorbidities such as stress, obesity and viral infections (2–4). Despite the healthcare advancements, asthma is still under-diagnosed and under-treated creating huge healthcare burden and social implications to individuals and concerned families (5). Also surprisingly, even after taking the existent therapy, a significant portion of the affected population continues to experience a recalcitrant form of asthma (6). This difficult-to-treat asthma is an attribute of either insensitivity to corticosteroids or poor therapeutic adherence due to various associated side-effects. Albeit the fraction of poorly controlled asthma subjects is relatively small, they form a major portion of mortality, morbidity and the associated cost.

The impetus behind this review is to accentuate the unmet dearth of newer therapy targets and to emphasize the need for customized therapeutics to bestir the challenges of current asthma therapy. The present article elaborates on asthma and the associated allies, a newer arena of fascinating drug targets – nuclear receptors (NRs) and how these could be instrumental in combating asthma and comorbidities.

## Asthma: Tale of an Atypical Ailment

The word ‘Asthma’ has Greek roots and its meaning translates to *short of breath*. However, it was in late 19^th^ century when the term was refined with the publication of a treatise entitled “On Asthma and its Treatment”. Thus, from that time, asthma came to be recognized as a distinct ailment which gets triggered by a specific set of stimuli and possesses clinical consequences (7). The modern definition as provided by Global Initiative of Asthma (GINA) describes asthma as a complex disease of lower airways characterized by bronchoconstriction, excessive mucus production, breathlessness and wheezing illness (8). The disease is a mixed outcome of genetic susceptibility and environmental influence resulting in a lifelong ailment (9). A consistent variability is also seen in disease expression in terms of provoking elements, age, gender, various forms of airway inflammation and index of severity. The most commonly observed form is of allergic asthma and its physiology involves T-cells, mast cells and eosinophils majorly along with histamine, cytokines and leukotrienes as inflammatory mediators (10). Asthma symptoms can range from mild to severe and hence, has been classified into various ‘phenotypes’ based on observable symptoms or environmental triggers. However, various phenotypes give no comprehension about the underlying molecular mechanisms and therefore, classification of asthma subtypes on the basis of involved cellular mechanisms led to the recognition of different ‘endotypes’ (11). They consist of distinctive pathophysiological features like T helper type 2 (Th2) associated allergic asthma, severe eosinophilic, allergic bronchopulmonary mycosis, aspirin-sensitive asthma, obesity-related asthma (lifestyle-linked) and non-Th2 linked neutrophilic asthma (12). However, the genetics behind most of these endotypes are not thoroughly deciphered and the variations in disease progression and severity remains largely unexplained.

**Figure 1:**
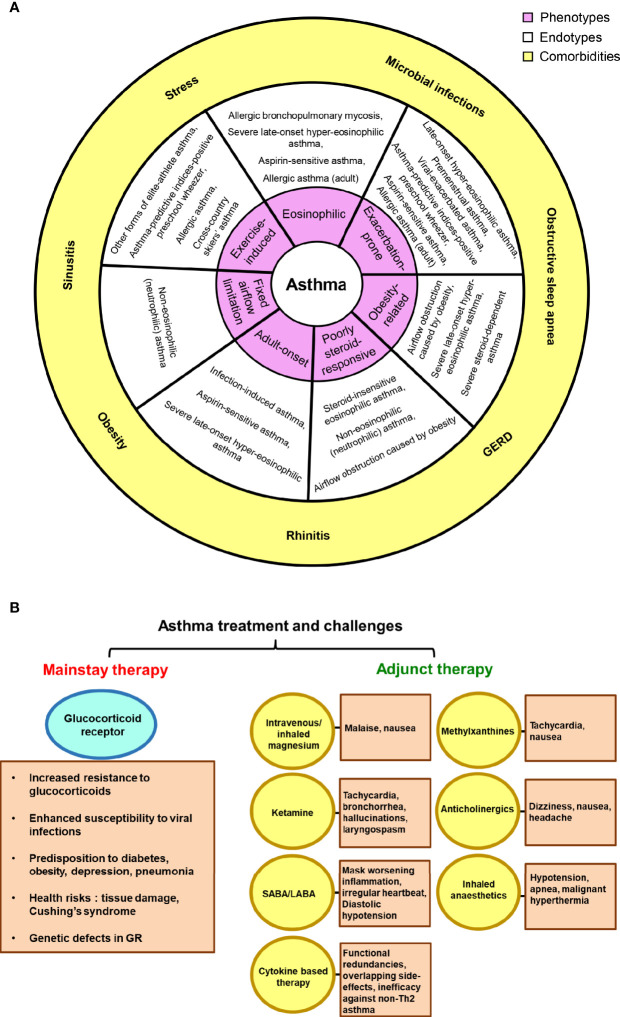
Asthma, comorbidities and treatment challenges. **(A)** The link between asthma phenotypes and endotypes is depicted; suggesting possible existence of multiple endotypes for a single phenotype and vice-versa. The outer sphere portrays the commonly observed comorbid conditions in asthma patients. **(B)** The reported side-effects and shortcomings of glucocorticoid receptor and other adjunct therapies in asthma have been highlighted.

Asthma exists with numerous comorbidities that mutually influence its clinical expression, disease management and control. Comorbid conditions like sinusitis, rhinitis, microbial infections, obstructive sleep apnea, psychopathologies, hormonal disturbances and gastroesophageal reflux disease (GERD) have been found to be highly prevalent (13). Also, demographic observations suggest of a possible hormonal angle and this has been considerably documented that women suffer more severe asthma and subsequent hospitalisations than men (14–17) (**Figure 1A**). The adult form of asthma witness significantly more comorbidities than the general asthmatic population with a ratio of one being found in every four adults. Although children had a lower comorbidity burden, yet 12.6% harbored a linked chronic medical condition (18). Further, asthmatics are shown to be more prone to arthritis, heart disease, diabetes, cancer, stroke and osteoporosis (19).

As recommended by the disease-management guidelines, inhaled corticosteroids and short-/long- acting β2-adrenoceptor agonists (SABAs and LABAs) form the cornerstone of current asthma treatment. *Salbutamol* and *terbutaline* hail to the SABAs category and are the most effective asthma relievers available till today. The two LABAs, *salmeterol* and *formoterol* are used as a supplementary therapy where patients do not experience relief with inhaled corticosteroids. These successfully induce bronchodilation for 12 h. However, LABA has the potential to mask worsening inflammation and hence, a monotherapy with LABA is not prescribed (20–22). Mounting evidence suggests that a significant fraction of asthmatics respond poorly to inhaled and systemic steroid therapy (23, 24). Treatment of glucocorticoid-resistant patients with GCs produces major health risks, ranging from irreversible tissue damage to the development of Cushing’s syndrome, thereby increasing the overall morbidity and mortality rates (25). Moreover, along with osteoporosis and pneumonia, oral corticosteroids may sway our body toward glucose intolerance/diabetes and also enhance susceptibility to viral infections (26, 27). The general non-compliance to GC treatment, genetic defects in GR and abnormal steroid pharmacokinetics further add to the issue (28, 29). Several other adjunct therapies are prevalent along with the mainstay that provide relief to severe asthmatic cases, such as methylxanthines, magnesium, anticholinergic drugs, ketamine or other inhaled anesthetics. However, these too, are gloomed by the associated dire effects (30–32). Furthermore, new and emerging biologic therapies for severe asthma are cytokine-based (anti-IL-4, IL-5, or IL-13) and mostly target Th2-mediated pathways. Nonetheless, they suffer from issues like functional redundancies and overlapping side-effects; thereby failing in clinical efficacy (33) (**Figure 1B**). At present, FDA has approved Omalizumab, a recombinant humanized anti-IgE monoclonal antibody for severe asthma, which down regulates the high-affinity IgE receptor FcϵR1 on mast cells and basophils (34). Since there is an absence of unifying markers for non-Th2 asthmatics, novel strategies to target this section of patients have yielded poorer success ratios than therapies for Th2 high asthma. Hence, the lack of an alternative effective therapy leads to high healthcare costs.

## Nuclear Receptors: A Clan of Idiosyncratic Transcription Factors

NRs hail from a large 48-membered ligand-dependent superfamily of transcription factors that are associated with the overall vertebrate development and are connected to a plethora of human diseases (35). The appeal of these receptors is that their activity can be regulated by binding to small lipophilic molecules called ligands (36). Ligands cross the lipid bilayer and carry out both intra- and extra-cellular physiological activities. The superfamily of NRs is typically grouped into three classes based on their ligand selectivity: class I, class II, and class III. The classical endocrine nuclear receptors catalog themselves to class I and comprise of estrogen (ER), glucocorticoid (GR), mineralocorticoid (MR), progesterone (PR), and androgen receptors (AR) as its members. Steroid hormones synthesized by endocrine glands serve as their ligands and these NRs bind to their DNA sequences in homodimeric fashion. NRs which get activated by dietary vitamins, importantly vitamin D receptor (VDR) and retinoic acid receptor (RAR) are also clubbed into this category, although they bind to DNA by forming heterodimers with other NRs. The ligand binding affinity is very high in such receptors due to a smaller ligand binding pocket. Class II corresponds to orphan NRs for which respective ligands have not been discovered so far. They bind DNA usually as monomer or homodimer and examples of such NRs include retinoid-related orphan receptors (RORs) and nerve growth factor IB (Nurr77). Class III represents adopted orphan NRs and comprise of those NRs which earlier belonged to orphan category but with recent discovery of their ligands, have constituted a new class. On comparison with endocrine NRs, these have larger ligand binding pockets and lower affinity for ligands. Peroxisome proliferator-activated receptors (PPARs), farnesoid X receptor (FXR), liver X receptor (LXR) and retinoid X receptor (RXR) are all examples of adopted orphan receptor (37, 38). The NRs largely share a conserved structure consisting of a DNA-binding domain (DBD), hinge region, ligand-binding domain (LBD) along with variable N-terminal and C-terminal domains. N-terminal is known for carrying out transactivation functions *via* activation function-1 (AF-1); independent of the ligand binding to the receptor. The DBD binds to the target DNA by recognizing certain conserved sequences known as response elements while hinge is a flexible region that connects DBD and LBD. The LBD is responsible for ligand based interaction and recruits coregulators through the activation function-2 (AF-2), which is followed by a C-terminal extension. NRs can bind to response elements of their target genes as homodimers, heterodimers and monomers and classically act as transcriptional repressors in the absence of a ligand. However, upon ligand binding, NRs undergo conformational changes that lead to release of corepressors, recruitment of coactivators and subsequent target gene activation (39). In addition to this, the class of adopted orphan receptors are more desirable as drug targets than endocrine receptors. Since adopted orphan receptors do not command the hormonal system, the adverse effects of immune and metabolic imbalance are averted. Moreover, orphan receptors also hold certain charm and curiosity because discovery of their ligands hold the potential of being used as new ‘modulatory switches’ of the human system.

In current years, NRs have been accentuated as strategic therapeutic targets due to their ability to translate nutritional and metabolic signals into gene regulation; significantly impacting human health and disease progression (40). Interestingly, the market share for drug targets also reveals the dominance of G-protein-coupled receptors (GPCRs), NRs and voltage gated ion channels, underlying their immense clinical importance (41, 42). Since NRs communicate directly through DNA response elements of their target genes and can also crosstalk with various other signalling pathways, the spectrum of advantages is considerably more. NRs are deep-seated inside the cells, have cleaner effector functions and exhibit specificity both at the DNA binding and ligand binding levels. As these ligand-dependent transcription factors glean their ligands from a varied set of fat-soluble hormones, vitamins and diet stemmed components, NRs provide us with a considerably better opportunity for functional modifications (35). In addition to this, modulation by synthetic ligands makes them the ideal target for ‘small molecule drugs’ family (43). As small molecules can be easily manipulated, drugs can be designed that are even more potent than the endogenous ligand, for example, dexamethasone which brings a greater biological response than cortisol (44). Due to the immense success of NRs as drug targets, discovering newer roles of NRs in diseases and designing agonist/antagonist for them appear as a promising research initiative. Therefore, it wouldn’t be a mendacious statement to say that NRs are the future of genomic medicine and would soon compete with GPCRs, ion-channels and kinases as ‘next-generation targets’.

## Nuclear Receptors in Asthma

**Figure 2:**
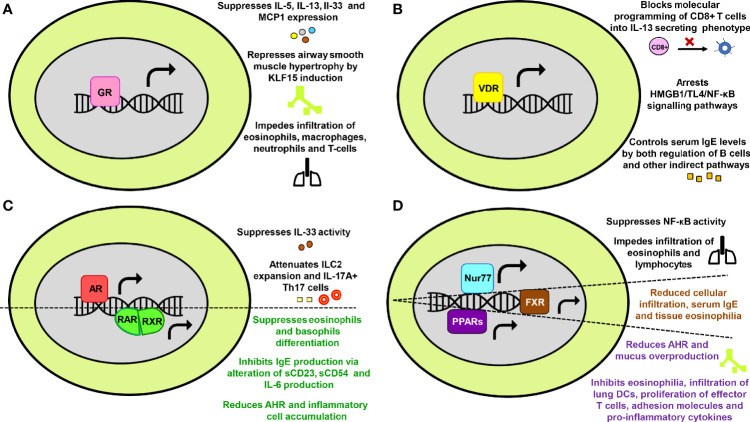
The protective effects of nuclear receptors (NRs) in asthma. Key mechanisms through which **(A)** Glucocorticoid receptor **(B)** Vitamin D receptor **(C)** Androgen receptor and Retinoic acid receptor-Retinoic X receptor **(D)** Nurr77, Farnesoid X receptor and Peroxisome proliferator-activated receptors provide protection against asthma have been summarized in a schematic form.

**Figure 3:**
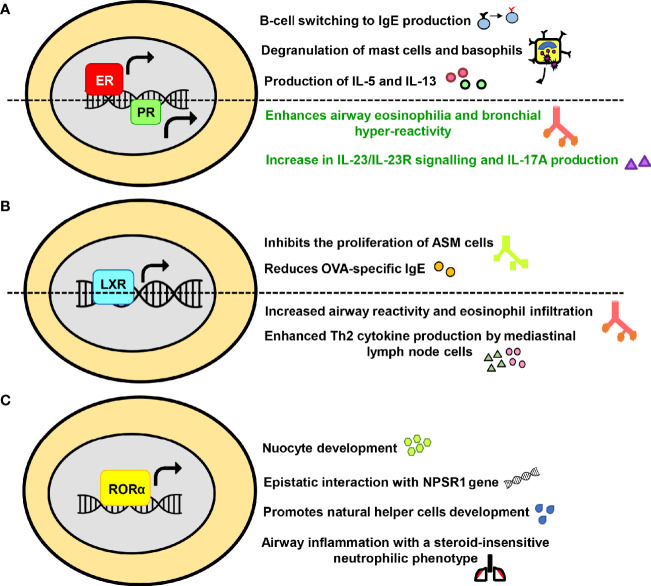
The exacerbating or promiscuous effects of nuclear receptors (NRs) in asthma. The influence of **(A)** Estrogen receptor and Progesterone receptor **(B)** Liver-X-receptor **(C)** Retinoic acid receptor-related orphan receptor alpha on major immunological pathways in asthma have been summarized in a schematic form.

With the recognition of NRs significance in physiology, they are now being extensively researched in numerous human diseases. In reference to asthma too, the accumulated data from scientific world stipulates the involvement of NRs in the pathology of asthma and its progression. Both kinds of NRs; those that abate asthma (**Figure 2**) and the ones that tend to promote the disease severity (**Figure 3**) are documented.

### Endocrine Receptors

#### Glucocorticoid Receptor

Glucocorticoids are among the most ubiquitous hormones present in mammals that carry out majority of their action through GRs. The GRs are involved in gluconeogenesis, postnatal insulin-like growth factor-1 production, behavioral abnormalities and physiological control of inflammation (45–51). In the context of asthma, GR is the most extensively studied NR. Glucocorticoids are known to have a suppressive effect on IL-5, IL-13, IL-33, and MCP1 expression, thereby impeding infiltration of eosinophils, macrophages, neutrophils and T cells into the lungs of asthmatic mice (52). GR also represses airway smooth muscle (ASM) hypertrophy in human ASM cells by inducing kruppel-like factor 15 (KLF15) (53). Furthermore, the GR expression levels or its subsequent phosphorylation are disrupted by multiple factors such as exposure to *Aspergillus fumigatus* (54), increased protein phosphatase 5 (PP5) activity (55), enhanced nuclear factor interleukin-3 (NFIL3) expression levels (56) and activation of H1R by histamine (57) (**Figure 2A**). Moreover, some recent reports have also linked gene polymorphism associated with GR as one of the plausible causes behind insensitivity to corticosteroid therapy (58, 59). Limited GR nuclear bioavailability (60, 61) and secondary effects of fetal sex and maternal asthma on placental GR expression are some of the other rationalities behind increased asthma susceptibility and exacerbations (62).

#### Vitamin D Receptor

Vitamin D earlier had its roots of significance among skeletal disorders such as osteoporosis, fractures, muscle strength, calcium, phosphorus and bone metabolism (63–66). However, research conducted in the past decade have established non-skeletal roles of vitamin D in chronic maladies including cardiovascular, infectious, metabolic, autoimmune diseases, cancer, and mortality (67–72). Vitamin D has been reported to arrest HMGB1/TLR4/NF-κB signaling pathway (73), inhibit the conversion of CD8+ T-cell to an IL-13 secreting phenotype through VDR recruitment to *Cyp11a1* promoter (74) and keep a check on serum IgE levels in B cells through both direct and indirect mechanisms (75) in asthmatic mice. In human B cells, IgE production, B cell proliferation and subsequent differentiation into IgE, IgG and IgA producing plasma cells are also inhibited by active form of vitamin D (1α,25-dihydroxyvitamin D(3)) *via* limiting the population of immunoglobulin‐secreting cells, reducing the activation of NF-κB and impeding switch recombination (76, 77) (**Figure 2B**). Moreover, factors like vitamin D deficiency and *VDR* gene polymorphism prevalent in human population have also been included in the ‘high susceptibility to asthma’ list (78–80).

#### Androgen Receptor

Primarily, AR plays a pivotal role in the development and maintenance of male phenotype (81). Along with sex-differentiation, fertility and male-specific pathology, AR is shown to modulate hair growth, immune functions, female reproductive growth, metabolic disorders and certain cancers (82–87). Since there is a visible sex bias in asthma, possible role of testosterone and other androgens in its immunopathogenesis have been studied. Gonadectomized male mice exhibited an increase in level of IL-13 protein expression, lymphocytes and eosinophils in comparison to the hormonally intact, sham-operated male mice (88). Another study has documented androgen-mediated inhibition of IL-33 driven lung inflammation and impaired expansion of group 2 innate lymphoid cells (ILC2) (89). Furthermore, stimulation of female mice with extract of *Alternaria alternata*, house dust mite (HDM) or ovalbumin (OVA) leads to increased production of IL-5 and IL-13 by ILC2 when compared with stimulated male mice (88–90). AR signalling has also been reported to directly attenuate IL-17A^+^ Th17 cells in the mice lung and indirectly reduces IL-13^+^ Th2 cells through impairment of HDM-induced IL-4 production (91) (**Figure 2C**). Since androgens also modulate airway hyperresponsiveness (AHR) and smooth muscle contractility (92, 93), AR signalling appears to play an important role in the regulation of allergic airway inflammation. However, a distinct role of AR in promoting M2 macrophage polarization has recently been revealed in a murine model of asthma (94). This finding perhaps suggests of a different role of AR in macrophages in contrast to other immune cells and therefore, requires further research.

#### Retinoic Acid Receptor and Retinoid X Receptor

The retinoid receptors comprise of two distinct subgroups, namely RAR and RXR that are known to modulate embryonic development, wound healing, neuronal differentiation, carcinogenesis, immunity and inflammation (95–98). Vitamin A serves as a ligand for both RAR and RXR, and provides shield against allergies by inhibiting IgE synthesis *via* alteration of sCD23, sCD54 and IL-6 production in anti-CD40 plus IL-4 stimulated human B cells (99). It also suppresses the differentiation of eosinophils and basophils at early stages of lineage commitment in HL-60 cells (human cell line) (100). Moreover, administration of RXR partial agonist NEt-4IB in mice has been found to significantly reduce AHR and inflammatory cell accumulation along with suppressing NF-κB expression (101) (**Figure 2C**).

#### Estrogen and Progesterone Receptors

Besides the effect on female reproductive system, estrogen and progesterone also significantly impact skeletal homeostasis, glucose and lipid metabolism, central nervous system and various cancers (102–107). Over the past decade, the role of female hormones in asthma, particularly estrogen, has been extensively recognized. The allergic responses are favored by estrogen in numerous ways such as fostering Th2 polarization, promoting B-cell switching to IgE production, enhancing mucus synthesis, increasing M2 polarization and assisting degranulation of basophils and mast cells (108–111). A couple of initial studies have reported that interaction of mast cells with estrogen enhances the release of histamines (112, 113). Other than histamine, estrogen mediated production of IL-5 and IL-13 from mediastinal lymph nodes has also been observed in an animal model of asthma (114). Moreover, it must be noted that exogenous compounds with estrogenic activity, such as xeno- and phytoestrogens interact well with ERs and are known to potentially influence histamine release and allergic responses (115–117). Additionally, an expression analysis has reported the expression of ER and PR in mast cells of human upper airways where they have been known to activate mast cells (118, 119). Differential expression of ER in asthmatics and receptor polymorphism have also been held accountable for elevating the asthma pathogenesis (120, 121). Interestingly, the role of ERβ differs from that of ERα and few recent studies have demonstrated its protective role in asthma *via* inhibition of PDGF induced proliferation, suppression of the NF-κB pathway, downregulation of AHR and airway remodeling (122–126). Furthermore, the impact of female hormone progesterone has been investigated in a murine model of asthma where it enhances airway eosinophilia and bronchial hyper-reactivity (127). The exposure to a combination of 17β-estradiol (E2) and progesterone (P4) also leads to an increase in IL-23/IL-23R signalling and IL-17A production in patients with severe form of asthma (128) (**Figure 3A**). Conceivably, the cardinal involvement of female hormones in asthma immunopathogenesis accounts for the observed gender disparity.

### Adopted Orphan/Orphan Receptors

#### Peroxisome Proliferator-Activated Receptors

The members of PPAR family are major regulators of energy homeostasis and metabolic function. However, broader roles have now been discovered and they are being increasingly recognized as key players involved in inflammatory, metabolic and neurodegenerative disorders (129). Experimental evidences have suggested that activation of PPARs produces anti-inflammatory effects in lung diseases (130). Deficiency of PPARα enhanced AHR and eosinophilia in a murine model of asthma while its agonist alleviated the disease (131–133). It is also reported that administration of PPARγ agonists or overexpression through adeno-PPARγ provides protection against asthma in mice (134–136). Several beneficial effects of PPARγ agonists in reducing multiple asthma features, such as AHR, leukocyte infiltration, mucus overproduction, infiltration of lung DCs, proliferation of effector T cells, adhesion molecules and pro-inflammatory cytokines have been observed in various murine asthma models (137–144). Troglitazone, another PPARγ agonist, impeded eosinophilia through inhibition of their IL-5 directed survival and eotaxin mediated chemotaxis (145) (**Figure 2D**). Collectively, the protective role of PPARs in lung disorders is affirmed by both experimental and clinical data (146–150). However, few recent animal based studies have instituted that PPARγ promotes type 2 effector responses in DCs and T cells (151, 152). Furthermore, therapy with PPARγ agonist (pioglitazone and rosiglitazone) showed modest or no improvement in subjects with asthma, thereby implying insufficient intervention (153, 154). Thus, rigorous studies and clinical research are required where a larger sample size and distinct endotypes are considered in order to translate PPARγ agonists into asthma drugs.

#### Nur77

The orphan nuclear receptor Nur77, also known as the nerve growth factor IB (NGFIB) is highly expressed in eosinophils, tolerant T cells and lung epithelium. It plays a cardinal role in mediating inflammatory responses in macrophages (155) and is also involved in cell growth, apoptosis, T cells function, neuronal regulation, muscle homeostasis and energy metabolism (156–164). Nur77 has been found to provide protection against asthma by suppressing nuclear factor kappa-light-chain-enhancer of activated B cells (NF-κB) activity. The knockdown of Nur77 led to enhanced inhibitor kappa B alpha (IκBα) phosphorylation and Nur77 knock-out mice had an increased infiltration of lymphocytes and eosinophils with aggravated mucus production. Moreover, bronchial hyper-responsiveness is also determined by single nucleotide polymorphism (SNP) present in *Nur77* gene (165) (**Figure 2D**).

#### Farnesoid X Receptor

The bile acid sensing receptor FXR largely controls hepatic triglyceride and glucose homeostasis (166). In addition to the above, its non-hepatic roles in fluid homeostasis, atherosclerosis, cancer, cardiovascular, renal, neuronal and other inflammatory diseases have also been documented (167–170). FXR is shown to have an immunomodulatory role in asthma and predominantly reduces the disease severity. Treatment with natural FXR agonists (chenodeoxycholic acid and ursodeoxycholic acid) significantly reduced cellular infiltration in peri-bronchial areas, mucus production, serum IgE and tissue eosinophilia in mice (171, 172) (**Figure 2D**).

#### Liver X Receptor

LXR finds its expression in a large number of cell types and is responsible for maintaining whole-body lipid and cholesterol metabolism (173). Newly discovered roles of LXR, linking lipid metabolism and inflammation, have also emerged; highlighting its importance in immune disorders such as infection, atherosclerosis and Alzheimer disease (174). There are several reports for the role of LXR in airway diseases and one of the earliest studies states that LXR agonist T1317 (T0901317) inhibited the proliferation of ASM cells (175). Similarly, agonist T0901317 provided protection in asthma *via* attenuation of OVA-specific IgE and reduction in collagen deposition and ASM thickness (176). However, LXR agonist GW3965 mediated enhanced airway reactivity that increased the growth of ASM in pre-clinical models of asthma is also documented (177). In addition to this, a recent study involving LXR-knockout mice have shown that eosinophilic airway inflammation and production of Th2 cytokines by mediastinal lymph node cells was abolished in the LXRα^−/−^β^−/−^ mice in both the OVA and HDM-induced asthma models (178). Furthermore, administration of LXR agonist GW3965 exhibited an increase in the eosinophilic airway disease and production of type 2 cytokines (178) (**Figure 3B**). Given the immunomodulatory role of LXR in asthma, these differential effects of ligands need to be adequately addressed.

#### Retinoid Acid Receptor-Related Orphan Receptor

RORα is functionally linked to have roles in circadian rhythm, immune regulation, cancer progression, neural development, cellular metabolism and autoimmune diseases (179, 180). Recently, RORα has been associated with allergic diseases as its expression level was found to be on the higher side in patients with therapy-refractory asthma along with *RORα* rs11071559C>T gene polymorphism accounting for elevated susceptibility to asthma (181–183). This finding is further affirmed by other reports that discuss the link of *RORα* SNPs with increased childhood asthma and exhibits an epistatic interaction with neuropeptide S receptor 1 (*NPSR1*), leading to the modification of joint risk effects (184). RORα has also been identified as a key factor required for ILC2 differentiation and is critically required for nuocyte development (185, 186). Furthermore, this NR is necessary for natural helper (NH) cells development and allergic inflammation (187). Few other animal studies have shown that RORα^sg/sg^ and RORγ^-/-^ mice exhibit a severely impaired allergic response to OVA (188, 189). Another member of the ROR family, RORγt, is reported to drive the allergic airway inflammation toward a steroid-insensitive neutrophilic phenotype (190). Inhibition of RORγt also led to suppression of allergic airway hyperresponsiveness and pulmonary neutrophilia in a murine model of asthma (191, 192) (**Figure 3C**).

## Nuclear Receptors at the Crossroads of Diet and Asthma: Key Molecules for Nutritional Targeting of Disease

Diet is considered as a major contributor to education and regulation of the immune system, particularly impacting systemic inflammation and generation of oral tolerance. Epidemiological cohort studies have showcased that diets which are rich in fruits and vegetables (e.g. Mediterranean diet) had lower allergic index while high maternal consumption of oily and processed foods enhanced the risk for allergies (193). In relation to asthma, high fat meals led to an increase in sputum neutrophils and several other genes regulating airway inflammation (194–196). On the other hand, reduction in airway neutrophil influx and IL-8 protein in nasal lavage was observed upon intake of fruits and vegetables (197, 198). The components of diet not only impact adult life but also influence the health of child during pregnancy. There are considerable evidences linking low maternal intakes of vitamins and fiber in pregnancy with increased risk of asthma development in children (199–202). Moreover, development of allergic airway diseases was also found to be inhibited by diet through significant regulation of the gut microflora composition (202–204).

Food components alter gene expression through NRs and epigenetic modifications; contributing profoundly to phenotypic plasticity and susceptibility to chronic diseases (205–207). Along with hormones, bile acid and sterols, several NRs are activated by components directly originating from food or drugs. Examples include vitamin A (RAR, RXR), vitamin D (VDR), fatty acids (PPAR), plant steroids (ER), and xenobiotic drugs (CAR, PXR) (208). Moreover, dietary components like curcumin are also known to cause histone modifications (acetylation/deacetylation) (209). Couple of studies have revealed high fat diet induced alterations in NRs expression and activity (210–212). Flavonoids, a category of plant secondary metabolites, also exert some of their effects through NRs and play a key role in dietary modulation of metabolism and linked diseases (213). Thus, NRs are cardinal players that influence both the genome and epigenome of an organism, thereby bridging this gap between genotype and phenotype. Since NRs effect on gene expression is sequence specific, identification of spatial arrangement present on binding sequence in food-bound NRs would provide a greater understanding of epigenetic modulation associated with a particular disease.

## Expert Opinion: Scientific Challenges and Future Perspectives

>The field of biology is marked by an extraordinary spatiotemporal interplay. NRs are exemplary in the same context as each NR regulates vast array of gene networks, distinct to cell type and origin. The breakthrough in the field of genetics, molecular and structural biology has edified our knowledge about the functional regulation of NRs. However, there are still innumerable unmet queries regarding the understanding of how dynamically these NRs govern transcriptional processes. One of the future goals could be: comprehending their interaction abilities with the genome, how they peddle chromatin remodeling and corresponding interactions with binding partners like other NRs or coregulators (enhancers/repressors). The physiological complexity that we see is perhaps, a reflection of the genomic complexity. Therefore, another key area that can be chased would be discovering factors that collaborate with NRs and aid them in carrying out tissue-specific roles. An understanding of their functional aspects would provide insights into various human physiological pathways and lead to the genesis of ‘customized and tailored’ therapeutics.

In the context of asthma too, scientific findings have pointed toward the existence of NRs that tend to promote or abate asthma. These discoveries fortify the importance of NRs in maintaining immune homeostasis. The enormous success of GR in inflammatory diseases is a well-acclaimed example of NR serving as an excellent druggable target. However, an important aspect arising out of the several studies is that the NRs like ER, AR or GR must be kept out of druggable zones. This is because they dictate the sensitive hormonal arm and if tinkered, would have a substantial impact on immune system, systemic inflammation and body homeostasis. Conceivably, this could be one of the reasons behind GR-linked dire effects and hence, it would be a sagacious policy to focus on the family of orphan NRs. Presently, the role of six orphan NRs; Nurr77, FXR, LXR, PPAR, ROR, and RXR have been reviewed in the context of asthma biology. Howbeit, the role of other members of this family is still encrypted and leaves an open canvas for future research. The complete landscaping of orphan NRs in immunopathogenesis of asthma would increase our understanding of how these NRs impact the complex gene networks behind various disease endotypes.

It is noteworthy to mention that many food ligands as well as drugs interact with NRs and activate them functionally. ERα ligand genistein, a food isoflavone, has been found to elevate the therapeutic efficacy of ERα antagonist tamoxifen in breast cancer (214). On the other hand, glucocorticoid-induced hyperinsulinemia was inhibited by PPARα agonist fenofibrate in mice fed on high-fat diet while enhancing the anti-inflammatory effects of GR on transrepression of NF-κB (215). Therefore, future efforts must be directed toward in-depth study of how these food-driven synergies and antagonisms influence NRs and the response to asthma therapy. In addition to this, role of gut microbiota in asthma and their regulation through healthy diet has gained much scientific attention. So, another interesting question to chase would be to decipher how the gut microbiota crosstalk through NRs and its impact on the gut microbiota-diet axis. However, before giving it a translational outlook, a comprehensive study elucidating the role of all 48 NRs in asthma and allergies needs to be done. Post the explication of their roles in current disease biology, alternative adjunct-therapies could be developed and chosen NRs could be targeted either *via* drugs (small-molecules) or dietary supplements. A combinatorial therapy of the FDA approved ligands; agonists for defender-NRs that possess anti-inflammatory attributes along with antagonist for disease-exacerbating NRs could be employed to relieve asthma in a synergistic way (43) (**Table 1**).

**Table 1:** List of nuclear receptors (NRs) and their effect on asthma pathogenesis along with information about natural/dietary and FDA approved ligands (agonists for protective NRs: green colored and antagonists for exacerbating NRs: red colored).

Nuclear Receptors	Nomenclature	Effect on asthma	Natural/Dietary Ligands	FDA approved ligands
**Glucocorticoid receptor (GR)**	NR3C1	**Reduce**	Glucocorticoids	Amcinonide, Prednisone, Mometasone furoate, Dexamethasone, Salmeterol, Budesonide, Hydrocortisone, Fluticasone propionate
**Vitamin D receptor (VDR)**	NR1I1	**Reduce**	Vitamin D, curcumin, lithocholic acid	Ergocalciferol, Cholecalciferol, Calcitriol, Paricalcitol, Alfacalcidol
**Androgen receptor (AR)**	NR3C4	**Reduce**	Androgens, diindolylmethane, epigallocatechin 3-O-gallate	Oxandrolone, Nandrolone phenpropionate, Testosterone, Danazol
**Retinoic acid receptor/Retinoic X receptor (RARα/RXRα)**	NR1B1/NR2B1	**Reduce**	Retinoic acid, lithocholic acid, phytanic acid	Tretinoin, Bexarotene, Adapalene, Acitretin, Tazarotene
**Peroxisome proliferator-activated receptor (PPARs)**	NR1C1, NR1C3	**Reduce**	Fatty acids, resveratrol, eicosanoids, arachidonic acid, genistein, daidzein, equol, tangeretin, nobiletin	Rosiglitazone, Pioglitazone, Metformin, Treprostinil, Balsalazide, Fenofibrate
**Nurr77**	NR4A1	**Reduce**	–	Not available
**Farnesoid X receptor (FXR)**	NR1H4	**Reduce**	Bile acids, epigallocatechin 3-O-gallate	Obeticholic acid, Chenodeoxycholic acid, Alpha-Linolenic acid, Ivermectin, Avermectin B1a
**Estrogen receptor (ER)**	NR3A1	**Enhance**	Estrogens, genistein, daidzein, equol, epigallocatechin 3-O-gallate, resveratrol	Fulvestrant (antagonist), Tamoxifen, Lasofoxifene (selective estrogen receptor modulators)
**Progesterone receptor (PR)**	NR3C3	**Enhance**	Progesterone	Mifepristone (antagonist), Ulipristal acetate (selective progesterone receptor modulator)
**Liver X receptor (LXR)**	NR1H3, NR1H2	**Selective modulator**	Oxysterols, epigallocatechin 3-O-gallate, genistein, ursolic acid	Not available
**Retinoic acid receptor-related orphan receptor alpha (RORα)**	NR1F1	**Enhance**	Sterols, ursolic acid	Not available

**Figure 4:**
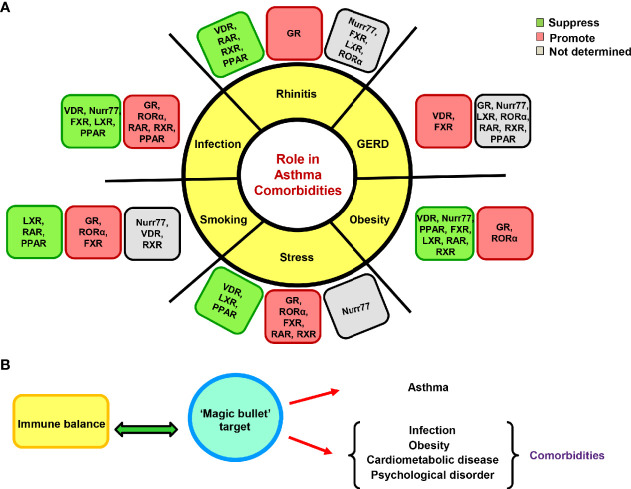
Effects of nuclear receptors (NRs) on asthma comorbidities. The NRs are also known to impact asthma comorbidities; both protective and exacerbating roles have been documented. **(A)** The illustration depicts the reported roles of GR and adopted orphan/orphan receptors in various comorbid conditions. **(B)** A proposed hypothetical model of a cleaner therapeutic target as a remedy for asthma and associated comorbidities that can also maintain immune homeostasis.

**Figure 5:**
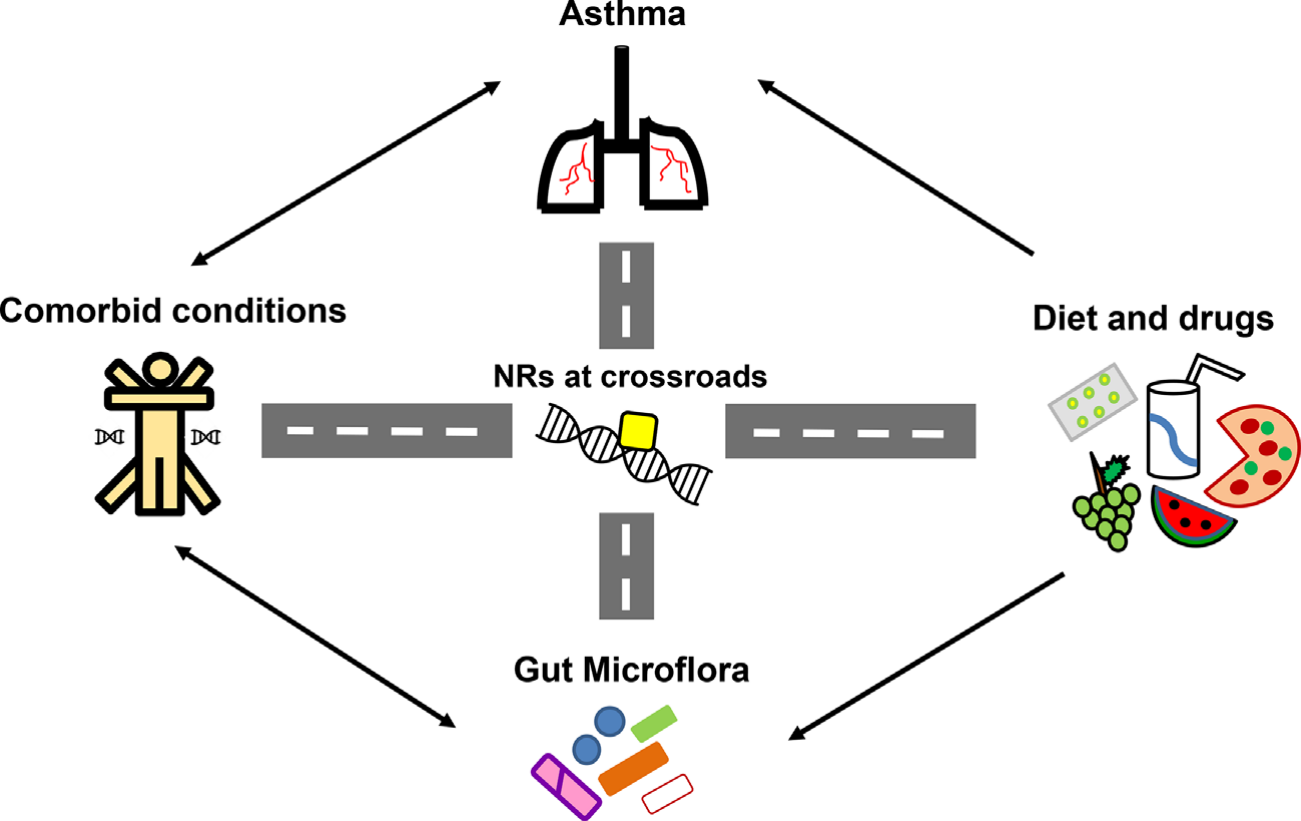
Nuclear receptors (NRs) at the crossroads of asthma, comorbidities, diet and gut microflora. Numerous studies have highlighted the significant influence of diet, gut microflora and existing comorbidities in asthma pathogenesis and therapy outcomes. With reported roles of NRs in asthma and comorbidities along with its ability to crosstalk with diet, drugs and microflora, NRs become the strategic molecules for therapeutic intervention in asthma.

The NRs annotated so far in asthma biology have their impacts on comorbidities as well. Whether its metabolic syndromes, microbial infections, cardiovascular or inflammatory disorders, all of these biological province corroborate either healing or augmenting roles of various NRs (216–220). With the cognition of available literature, it becomes apparent that GR is an odious target for the treatment of refractory asthma and comorbid conditions as both aberrant GR expression and gene polymorphism is found to be associated with several metabolic, inflammatory and psychiatric illnesses. The GR has been reported to promote adipogenesis, obesity, musculoskeletal disorders, and psychosocial stress along with reduction in body’s ability to clear infection (27, 221–229). Another NR, RORα has similar attributes and it exacerbates both asthma and associated comorbidities (230–235). On the other hand, NRs like VDR and Nurr77 seem to be promising in their action and allay asthma, rhinitis, obesity, infection, stress or other co-existing conditions (236–245). Although Nurr77 is a desirable target but is considerably less explored and requires more translational studies. However, PPARγ, FXR, RXR, RAR, and LXR have multifarious effects where they mitigate few while exacerbates other comorbidities (101, 246–287). However, they can be critically chosen for creating a therapeutic cocktail in context of subjects exhibiting a particular comorbid condition along with asthma, thereby opening customized therapy avenues. Also, for a broader effect on clinical symptoms, there is a necessity to empower defender NRs as well as to countervail the disease advocating NRs. Therefore, a better remedy option would comprise of respective agonists of VDR and Nurr77 along with antagonist of RORα for its neutralization. Before advancing toward designing therapeutic cocktails, there is an inevitable need to evaluate the role of all 48 NRs in both asthma and comorbid conditions. Since asthma manifestation requires a genetic pre-disposition and fomenting environment, a better comprehension of the genetic basis would certainly speed-up the journey of finding new targets and subsequent drug development. These efforts would aid in the discovery of a cleaner target that might soothe both asthma and associated comorbidities (**Figures 4** and **5**).

## Conclusion

- There is no skepticism associated with the intake of a balanced diet and proper lifestyle patterns leading to a healthier immune system. Hustle and bustle of earning the daily bread and raised living standards have led to poor health quality and increased allergy burden. This review highlights the unmet dearth of newer therapy targets and elaborates on asthma therapy challenges like GR-associated side-effects and resistance issues along with a therapy need for linked comorbidities. It also discusses the cardinal importance of NRs in maintaining immune homeostasis and provides a knowledge window of how they serve as excellent druggable targets. Additionally, the advantages of adopted orphan NRs over endocrine NRs and why they should be the cynosure of all pharmacological efforts is discussed.
- The diets rich in fibers and micronutrients involving essential vitamins like A or D provide a natural defense against allergies while those that are calorie rich raise the vulnerability index. Taken together, these discoveries propose a possible future employment of patient-friendly therapy involving a combination of dietary supplements and synthetic ligands that would trigger the activation of defender NRs.
- The extent to which this perception of comorbid conditions worsening asthma holds true, the similar amount of veracity retains in the fact that asthma too can induce or even worsen comorbidities. They either share similar pathophysiological pathway (e.g. rhinitis) or alter asthma phenotypes (e.g. obesity, smoking, respiratory infections). Therefore, the apprehension and medication of comorbidities must be considered essential during the course of asthma evaluation. Considering the NRs significance in both asthma and comorbidities, there is a requisite to engage defender NRs alongside the negation of exacerbating NRs for a better cure. Moreover, mysteries to the underlying molecular link connecting asthma to these specific comorbid ailments still remain unresolved and finding answers to them would bring about a cut above understanding of disorders like asthma that display complicated etiology. The application of systems biology and findings from holistic studies would help in uncovering cleaner targets that are more desirable and possess some kind of ‘magic-bullet’ properties.

## Author Contributions

PG conceived the idea. DT and PG wrote the review manuscript. All authors contributed to the article and approved the submitted version.

## Funding

This work was supported by the Council of Scientific and Industrial Research (CSIR) 12th Plan Network project Bugs to Drugs (BSC0211) and OLP (0704) to PG.

## Conflict of Interest

The authors declare that the research was conducted in the absence of any commercial or financial relationships that could be construed as a potential conflict of interest.

## Abbreviations

AR, androgen receptor; ASM, airway smooth muscle; ER, estrogen receptor; FXR, farnesoid X receptor; GR, glucocorticoid receptor; LXR, liver X receptor NR, nuclear receptor; PPAR, peroxisome proliferator-activated receptor; PR, progesterone receptor; RAR, retinoic acid receptor; ROR, retinoid-related orphan receptor; RXR, retinoid X receptor; Th2, T helper cells type 2; VDR, vitamin D receptor.
